# Oral Proliferative Verrucous Leukoplakia: Progression to Malignancy and Clinical Implications. Systematic Review and Meta-Analysis

**DOI:** 10.3390/cancers13164085

**Published:** 2021-08-13

**Authors:** Gaspare Palaia, Amelia Bellisario, Riccardo Pampena, Roberto Pippi, Umberto Romeo

**Affiliations:** 1 Department of Oral and Maxillofacial Sciences, “Sapienza” University of Rome, Via Caserta 6, 00161 Rome, Italy; gaspare.palaia@uniroma1.it (G.P.); roberto.pippi@uniroma1.it (R.P.); umberto.romeo@uniroma1.it (U.R.); 2 Centro Oncologico ad Alta Tecnologia Diagnostica, Azienda Unità Sanitaria Locale—IRCCS di Reggio Emilia, 42123 Reggio Emilia, Italy; riccardo.pampena@ausl.re.it

**Keywords:** proliferative verrucous leukoplakia, oral cancer, biopsy

## Abstract

**Simple Summary:**

Proliferative verrucous leukoplakia (PVL) was recognized in 2005 by the World Health Organization as a rare subtype of true oral leukoplakia, with unknown etiology. Since its first description in 1985, several diagnostic criteria have been proposed over the years. The aim of this systematic review is to investigate the percentage of patients diagnosed with proliferative verrucous leukoplakia who progressed to oral verrucous carcinoma (OVC) or conventional oral squamous cell carcinoma (OSCC).

**Abstract:**

Aim: The aim of the present systematic review was to investigate the risk of malignant transformation of proliferative verrucous leukoplakia (PVL). Materials and Methods: the search was carried out using a combination of terms (leukoplakia OR leucoplakia) AND (multifocal OR proliferative) on the following databases: PubMed, Scopus, Web of Science (WOS Core Collection), Cochrane Library, selecting only articles published since 1985 and in the English language. Demographic, disease-related, and follow-up data extracted from the studies included in the qualitative synthesis were combined. Weighted means ± standard deviations were calculated for continuous variables, while categorical variables were reported as frequencies and percentages. Dichotomous outcomes were expressed as odd ratios (ORs) with 95% confidence intervals (CIs). Odd ratios for individual studies were combined using a random-effects meta-analysis, conducted using Review Manager 5.4 Software (Cochrane Community, Oxford, England). Results: twenty-two articles were included, with a total of 699 PVL patients, undergoing a mean follow-up of 7.2 years. Sixty-six percent of patients were females, with a mean age of 70.2 years, and 33.3% were males, with a mean age of 59.6 years. Most patients were non-smokers and non-alcohol users, and the gingiva/alveolar ridge mucosa was the most involved anatomical site by both PVL appearance and malignant transformation. A total of 320 PVL patients developed oral verrucous carcinoma (OVC) or conventional oral squamous cell carcinoma (OSCC) because of malignant transformation of PVL lesions (45.8%). A statistically significant 3.8-fold higher risk of progression to conventional OSCC was found compared to OVC in PVL patients, with women being 1.7 times more likely to develop oral cancer than men, as a consequence of PVL progression. Moreover, a statistically significant higher likelihood of developing conventional OSCC in female PVL patients than in males was found. In 46.5% of patients with PVL malignant transformation, multiple carcinomas, in different oral sites, occurred during follow-up. Conclusions: PVL is an aggressive lesion, which, in a high percentage of cases (almost 50%), undergoes malignant transformation, mainly toward OSCC. The female gender is most affected, especially in the elderly, with a negative history for alcohol and tobacco consumption.

## 1. Introduction

Proliferative verrucous leukoplakia (PVL) was recognized in 2005 by the World Health Organization (WHO) as a rare subtype of true oral leukoplakia, with unknown etiology, which mainly affects women in old age, often with a clinical history that does not include tobacco or alcohol consumption, with high risk of malignant transformation and diagnosis, usually retrospective, based on the association of clinical and histopathological aspects [1].

The expression “proliferative verrucous leukoplakia” was firstly introduced in literature in 1985 by Hansen et al. to define a lesion characterized mainly by multifocal presentation, exophytic and verruciform appearance, resistance to all therapeutic approaches, both surgical and non-surgical, high tendency to malignant transformation and progressive histological changes, evidenced in sequential biopsies, with evolution from simple hyperkeratosis, to lesions with increasing degrees of dysplasia, oral verrucous carcinoma (OVC), and conventional oral squamous cell carcinoma (OSCC) [2].

Several diagnostic criteria for PVL have been proposed over the years to help clinicians in diagnosis. In 2010, Cerero-Lapiedra et al. identified some major and minor criteria [3], which were then eliminated in 2013 by Carrard et al. [4].

Finally, in 2018, Villa et al. suggested that all the following criteria must be satisfied for a lesion to be classified as a PVL [5]:

(1) White/keratotic lesions that can be smooth, fissured, verrucous, or erythematous, with or without the presence of ulcerated areas.
(2) Non-contiguous multifocal lesions or single lesions larger than 4 cm in a single site or single lesions larger than 3 cm with the involvement of contiguous sites.
(3) Lesions that progress/expand and/or develop multifocality over time.
(4) Histopathology, which, in absence of dysplasia or carcinoma, still shows hyperkeratosis, parakeratosis, atrophy, or acanthosis with minimal or absent cytological atypia, with or without the presence of a lymphocytic band, or verrucous hyperplasia.

The most recent guidelines about the management of the proliferative verrucous leukoplakia include constant follow-up, every 3–6 months, with biopsies involving new onset red, or nodular areas, and areas of increased consistency [5].

Proliferative verrucous leukoplakia belongs to oral potentially malignant disorders [6] with the highest rate of neoplastic progression. Therefore, the aim of this review is to investigate the percentage of patients diagnosed with PVL who, over the years, underwent the development of OVC or OSCC.

## 2. Materials and Methods

**Figure 1:**
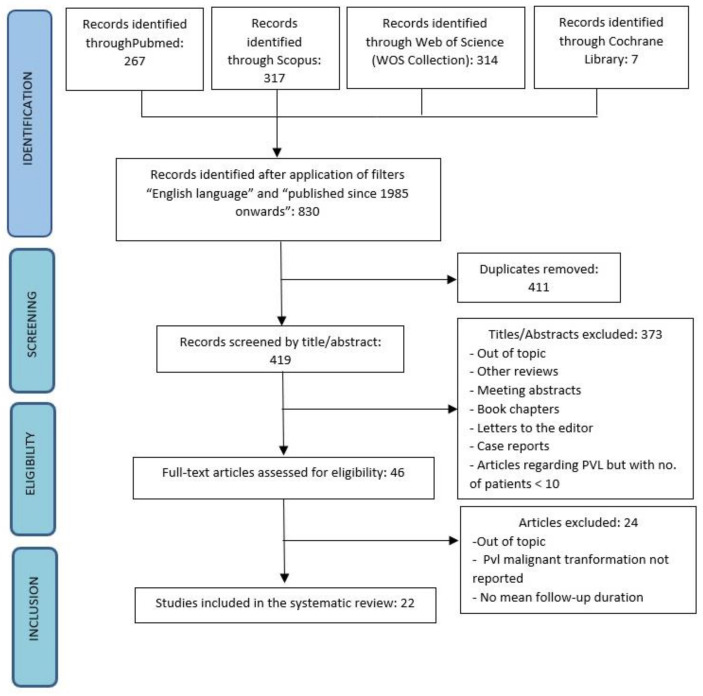
Flowchart of search results and study selection.

A systematic review was conducted according to the Preferred Reporting Items for Systematic Reviews and Meta-Analyses (PRISMA) guidelines (Figure 1) and the Meta-Analysis of Observational Studies in Epidemiology (MOOSE) proposal when applicable. The protocol was registered on the international prospective register of systematic reviews (PROSPERO), with the reference number CRD42021229215.

The primary outcome was the ratio of PVL patients progressing to oral cancer (OSCC vs. OVC), while secondary outcomes included progression to cancer among genders.

The search was carried out using a combination of terms (leukoplakia OR leucoplakia) AND (multifocal OR proliferative) on the following databases:

− PubMed.
− Scopus.
− Web of Science (WOS Core Collection).
− Cochrane Library.

Only articles published in English from 1985 to 26 November, 2020, were selected.

The inclusion criteria for articles to be included were:

− Studies involving at least 10 PVL patients.
− Diagnosis of PVL carried out on the basis of the criteria defined by Hansen et al. [2], or subsequent modifications.
− Studies reporting the number of patients who, over time, developed oral cancer as a consequence of PVL neoplastic transformation.
− Articles in which the mean follow-up of PVL patients was explicitly reported.

The following sources were excluded from this review: other reviews, meeting abstracts, letters to the editor, book chapters (Figure 1).

The literature search was performed independently by two researchers (A.B., R.P.) and, in case of disagreement, the intervention of a third researcher (G.P.) was requested.

During the article selection procedure, in case of suspicion of patients included in more than one study, an email was sent to the authors to receive detailed information; in case of a missing answer, or whether it was not possible to identify any duplicates, it was decided to include, in this review, only the most recent article with the greatest number of cases. At the end of the search procedure, 22 articles were included in this systematic review (Table 1).

Before proceeding with the processing of results, a table was created in Microsoft Excel, in which data obtained from individual studies were entered.

The information considered for each article included:

- First author;
- Year of publication;
- Country in which the study was conducted;
- Enrolment period of patients;
- Type of study with any statistical analysis applied;
- Number of patients with PVL diagnosis;
- Gender;
- Mean age;
- Mean duration of follow-up;
- Current or past use of tobacco or alcohol;
- Number of patients who underwent neoplastic transformation of the lesion, including the number of men and women;
- Subjects who developed verrucous carcinoma or conventional oral squamous cell carcinoma (included gender);
- Number of patients who developed multiple carcinomas in different intraoral sites;
- Mean time from diagnosis of PVL to neoplastic transformation;
- Mean number of biopsies per patient;
- Mean time between first and eventual second primary oral carcinoma;
- Number of patients who died from causes related to oral carcinoma;
- The site most frequently involved by the onset of PVL or by malignant transformation.

Demographic, disease-related, and follow-up data extracted from the studies included in the qualitative synthesis were combined. Weighted means ± standard deviations were calculated for continuous variables, while categorical variables were reported as frequencies and percentages.

Dichotomous outcomes were expressed as odd ratios (ORs) with 95% confidence intervals (CIs).

Odd ratios for individual studies were combined using a random-effects meta-analysis.

The meta-analysis was conducted using Review Manager 5.4 Software (Cochrane Community, Oxford, UK).

The evaluation of Recommendation and Quality of Evidence of included studies was conducted according to Robinson et al.’s assessment [27] (Table 2).

## 3. Results

Twenty-two articles were included. One of them [12] referred to the same patient group reported by another [11], but provided data related to the development of multiple carcinomas (Table 1). Overall, the total number of patients with a diagnosis of PVL was 699. The mean age, reported in 19 studies, involving a total of 573 patients [2,5,7,8,9,10,11,13,14,15,16,17,18,19,20,21,22,23,25], was 64.2 ± 10.5 years, while follow-up had a variable duration, from 3.3 to 16 years (mean value 7.2 ± 6.3 years) (Table 3).

The percentage of male and female subjects, reported in 20 articles [2,5,7,8,9,10,11,13,14,15,16,17,18,19,20,21,22,23,25,26], was, respectively, 33.3% (208 out of 624) and 66.7% (416 out of 624), with a mean age of 59.6 years for men and 70.2 years for women [2,5,7,8,10,14,17,18,20,21,23,25,26] (Table 3).

There were 11 studies reporting the enrolment period of patients [2,5,10,13,16,18,19,20,21,22,24], ranging from 4 to 31 years, with a mean value of approximately 20 years, and a range from 1985 to 2020, although in most articles, the type of enrolment (consecutive or non-consecutive) was not reported.

Out of the 699 patients, 106 were from California (USA) [2,8,9,10], 189 from United Kingdom [7,19,20,26], 174 from Spain [11,14,16,18,22,25], 122 from Italy [13,24], 15 exclusively from Brazil [17], 21 from Brazil and Guatemala [15], 20 from Florida (USA) [21], 10 from Georgia (USA) [23] and 42 from Massachusetts (USA), New York State (USA), and Brazil [5].

Current or past tobacco use (smoked or chewed) was reported in 19 studies, for 218 out of 544 PVL patients (40.0%) [2,5,7,8,9,10,11,13,14,15,16,17,18,19,21,22,23,25,26] (Table 3), while eight studies [5,13,15,17,18,19,25,26] described the spread of consumption of alcohol, which turned out to be 26.2% (65 out of 248 PVL patients).

Avoiding considering the studies that selected only PVLs with gingival localization [10,22], there were 16 articles which reported the lesion site [2,5,7,8,11,13,15,16,17,18,19,20,21,23,25,26]. The most involved site was the gingiva/alveolar ridge mucosa, followed, in equal measure, by the tongue and the buccal mucosa, and finally the vestibule.

The number of patients undergoing PVL malignant transformation during the follow-up, with the development of at least one oral carcinoma, was 320 (45.8%; Table 3).

**Figure 2:**
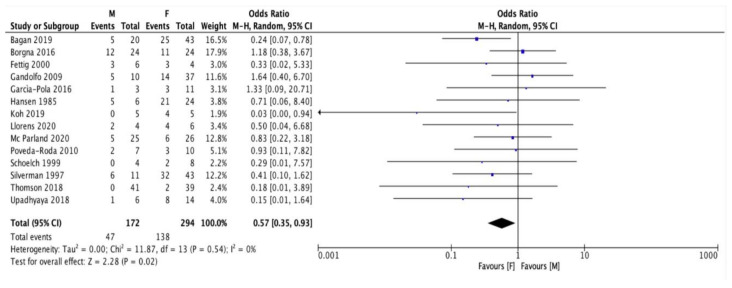
Forest plot relating to the prevalence of neoplastic events in male (M) and female (F) patients.

A statistically significant difference was found in the probability for female vs. male patients to undergo malignant transformation of PVL, with women being 1.7 times more likely to develop cancer as a consequence of PVL progression than men (OR 0.57, 95% CI 0.35–0.93) (Figure 2).

**Figure 3:**
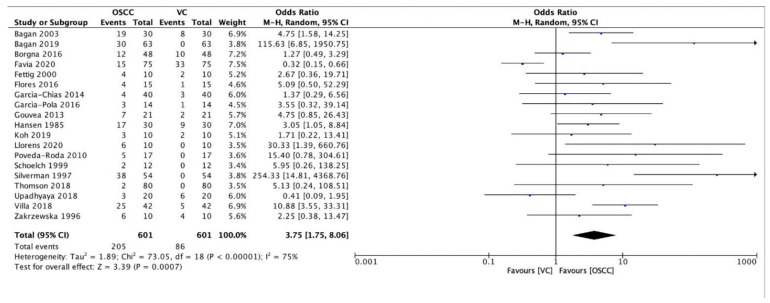
Forest plot relating to the distribution of conventional oral squamous cell carcinomas (OSCC) and verrucous carcinomas (VC) among patients with PVL.

In addition, a statistically significant 3.8-fold higher risk of progression to conventional squamous cell carcinoma (OSCC) compared to verrucous carcinoma (OVC) was found in PVL patients (OR 3.75, 95% CI 1.75–8.06) (Figure 3).

**Figure 4:**
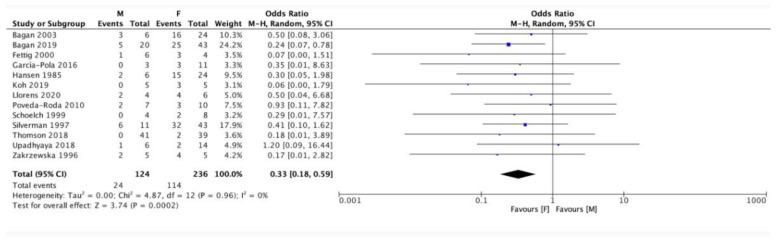
Forest plot relating to the distribution of conventional oral squamous cell carcinomas (OSCC) in male (M) and female (F) patients.

The likelihood of developing OSCC was found to be approximately three times higher in female PVL patients than in male PVL patients, which was statistically significant (OR 0.33, 95% CI 0.18–0.59) (Figure 4).

**Figure 5:**
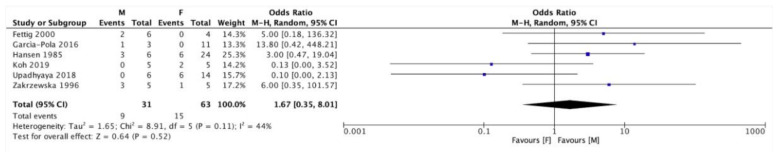
Forest plot relating to the distribution or oral verrucous carcinomas (OVC) in male (M) and female (F) patients.

On the other hand, it seemed that the development of an OVC was more likely in men than in women, even if in this case no statistical significance was found (Figure 5).

There were 12 articles that reported the time elapsed between the diagnosis of PVL and its malignant transformation, with a mean value of 4.1 years [5,8,9,10,11,13,16,18,19,21,22,25].

In 87 out of a total of 187 patients with malignant transformation of PVL (46.5%; Table 3) [5,8,12,13,14,15,17,21,24,25], multiple carcinomas were diagnosed in different oral sites during the follow-up.

Only three studies revealed the average time elapsed between the development of the first and a possible second primary cancer, with values of 1.6 [11], 1.81 [13], and 1.5 [24] years.

The mean duration of follow-up for PVL patients without malignant transformation was 5.8 years; for those with malignant transformation, it was 8.3 years [2,5,13,17,19,21,23,25].

Regarding the most involved site by malignant transformation, excluding studies selecting only gingival PVLs [10,22], in seven studies, the gingiva/alveolar ridge mucosa was the most frequently affected site [8,12,13,18,21,24,25].

Six studies, for a total of 160 subjects, provided information about the number of biopsies performed in each patient, with a mean value of 7.5 biopsies per patient [2,5,7,19,21,23].

Regarding the survival of PVL patients during follow-up, data related to patients who died because of the development of one or more oral cancers from a pre-existing PVL was provided by nine studies: 40% of patients with a mean follow-up of 6.1 years [2], 20% with a mean follow-up of 6.6 years [7], 38.9% with a mean follow-up of 11.6 years [8], 10% with a mean follow-up of 4.4 years [10], 5.2% with a mean follow-up of 5.1 years [12], 0% with a mean follow-up of 5.4 years [17], 14.3% with a mean follow-up of 14.5 years [18], 12.5% with a mean follow-up of 4.3 years [19], and 5.3% with a median follow-up of 5.2 years [24].

Combining the information provided by the various studies, 49 out of 286 patients died as a consequence of oral cancer developed from PVL (17.1%).

Concerning quality assessment, all of the included studies were evaluated as reporting weak recommendation and limited-quality, patient-oriented evidence, independently from the study design (case series, cohort, case-control). The low quality of evidence on this topic, amplifies the need for a meta-analysis as well as for further prospective studies.

## 4. Discussion

In regard to sex distribution of PVL, according to the literature [28,29,30], women were involved by PVL more (416 = 66.7%) than men, especially in old age (mean age: 70.2 years). The gingiva/alveolar ridge mucosa was found to be the most involved intraoral site by both PVL and its malignant transformation. Usually, the lesion first involves the gingiva of a single tooth, often buccally, and then extends, over time, to that of the adjacent teeth, also on the lingual and palatal sides, likely involving the interdental periodontal tissues [5]. Therefore, any gingival leukoplakia, which progressively involves other anatomical sites over the years, must be viewed with suspicion. McParland and Warnakulasuriya in 2020 reported that 19 of the 51 patients with PVL had gingival lesions at the time of the disease onset (37.2%), while, at the last follow-up, gingival involvement was found in 33 out of the 51 patients (64.7%). Similarly, among patients who underwent neoplastic evolution of PVL, most cancers arose at the gingival level [26]. Bagan et al. in 2011, in a retrospective study on 55 subjects with PVL, found that the gingiva was the most frequently involved site by PVL (89.1% of cases), and that more than 50% of the OSCCs developed from gingival lesions [31]. Silverman and Gorsky also reported that PVLs of the gingiva, as well as those of the tongue, had a higher tendency to malignant transformation (*p* = 0.13), compared to those of all other oral sites [8].

Regarding the recurrence of gingival PVL, Villa et al. [5] hypothesized that it was correlated not only to clinical features, but also to a possible incomplete excision of pathological tissue, at the level of crevicular epithelium, which therefore was able to repopulate the biopsied area, leading to the progression of residual pathological cells, rather than to a real recurrence. There are only two studies describing the tongue as the most affected site of PVL malignant transformation [15,17].

The percentage of patients with malignant transformation of PVL, in the selected studies, varied from 2.5% to 100%, with a mean value of 45.8% (320 out of 699 subjects), and a mean follow-up of 7.2 years.

Women have a significantly higher risk of developing cancer from a previous PVL than men (OR 0.57, 95% CI 0.35–0.93), even considering the higher incidence of PVL in females. Only Borgna et al. [19] found a slight opposite prevalence between men and women (12:11) among PVL-related cancer patients.

In the present review, a statistically higher risk of PVL transformation to OSCC rather than OVC was found, with articles by Favia et al. [24] and Upadhyaya et al. [21] being the only exceptions. However, the risk that a patient with PVL may develop verrucous carcinoma is still higher than in the general population since the prevalence of oral verrucous carcinoma varies from 2% to 12% [32,33], while the present study found a 29.5% prevalence (86/291) in PVL patients.

It was also found that females not only have a higher incidence of PVL with an increased risk of its malignant transformation, but they also have a statistically higher risk of PVL progression to OSCC than males.

On the other hand, the occurrence of a verrucous carcinoma seems to be more likely in men, although with no statistical significance; thus, males, in addition to being less affected by both PVL and its malignant transformation, seem to be more frequently involved by OVC rather than OSCC, which has a much lower aggressiveness than OSCC [34], although further investigation is needed to verify this datum.

It is worthy of note that patients with PVL who develop an oral cancer, in 46.5% of cases develop at least one second tumor in a different intraoral site. In this regard, Bagan et al. [35] published a retrospective comparative study between a group of 33 patients with two or more PVL-related OSCC and a group with 48 no-cancer PVL patients, and found that clinical factors associated with the possibility of belonging to group 1 were a longer follow-up and a greater number of PVL-affected oral sites. Furthermore, the time interval between the diagnosis of each carcinoma and the next was, each time, shorter, and the most involved anatomical site by the first four carcinomas was gingiva, while buccal mucosa or the tongue were more frequently involved by the fifth new carcinoma [35].

The multifocal presentation typical of PVL and the high risk of developing multiple primary oral carcinomas have been associated with the concept of field cancerization, proposed in 1953 by Slaughter et al. [36], and subsequently suggested by others [37,38], to explain the occurrence of more than one carcinoma in the same district. This theory argues that there is the presence of genetically mutated cells even beyond the area with alterations evident on clinical or histopathological examination and the main molecular alterations would involve mutations in oncogenes or tumor suppressor genes, genomic instability and loss of heterozygosity. In the case of the upper aero digestive tract, these alterations have usually been correlated with prolonged exposure to carcinogens, such as tobacco; however, since most patients with PVL are not tobacco users [2,8], probably further, but yet unidentified, mechanisms may be involved. In this regard HPV infection has also been suggested having a role both in PVL etiopathogenesis and in its malignant transformation [39,40]. Moreover, PVL has different demographic aspects than those of conventional true oral leukoplakia [28] and, frequently, a verrucous appearance, which is typical of HPV-related lesions. However, other authors did not find any statistical correlation between HPV infection and both PVL [41,42] and its progression to malignancies [21]; therefore, it is not possible to exclude or confirm a possible etiopathogenetic role of HPV.

The consumption of tobacco (smoked or chewed) and alcohol does not seem to play an important role in the onset of this lesion, differently from the true leukoplakia, for which tobacco is recognized as the only risk factor [43]. Collecting data from the individual studies analyzed, a current or previous tobacco consumption was found in 218 out of 544 patients with PVL (40%), and alcohol use in 65 out of 248 subjects (26.2%). In five studies, the number of tobacco users exceeded half of the analyzed subjects [2,7,19,21,23]. Moreover a significant number of patients with PVL malignant transformation were not smokers; Bagan et al. in 2003 reported the presence of 4 tobacco users among 19 with PVL and progression to OSCC (21.1%) and 78 among 110 patients with OSCC not preceded by PVL lesions (70.9%) [11]. Favia et al., described the presence of 11 smoking patients among 48 with PVL who developed at least one carcinoma (22.9%) [24]. Borgna et al., in 2016, found no significant differences regarding tobacco and alcohol use between the 23 patients with PVL and malignant transformation and the 25 individuals with PVL without malignant transformation [19].

As for the etiopathogenesis of PVL-related carcinomas—in 2015, Akrish et al. hypothesized [44] that PVL-related OSCC represented a distinct entity with respect to PVL-unrelated OSCC since many features differed in the two conditions. They found PVL-related OSCC being more frequently featured by the following features: early stage of development, small tumor size, no lymph node metastases, gingiva and buccal location, local relapses or second primary tumor, and a good 4-year survival rate (100%). On the contrary, PVL-unrelated OSCC were featured by the following features: more advanced stage of development with wider tumor size, high frequency of lymph node metastases (36.7%), lingual margin and mouth floor mucosa as prevalent locations, less frequent relapses or second tumors (12.2%). This hypothesis, obviously, may have practical implications in choosing both treatment strategies and follow-up timing for OSSCs occurring in the two different conditions, although further comparative studies with wider sample sizes are needed to corroborate such a suggested point of view.

## 5. Conclusions

According to the results of this systematic review, it can be stated that PVL is an aggressive lesion, which, in almost 50% of cases, undergoes malignant transformation, mainly toward OSCC. Women are more involved than men, especially in older age and with a negative history of alcohol and tobacco consumption.

Early diagnosis and constant surveillance with periodic biopsies are of paramount importance in management of PVL patients, especially when an oral carcinoma has already developed.

## Figures and Tables

**Table 1:** Studies included in this systematic review.

Authors	Year of Publication	Number of PVL Patients	Patients Enrolment Period	Type of Study
Hansen et al. [2]	1985	30	1961–1983	Retrospective case series
Zakrzewska et al. [7]	1996	10	-	Case series
Silverman e Gorsky [8]	1997	54	-	Case series
Schoelch et al. [9]	1999	12	-	Case series
Fettig et al. [10]	2000	10	1995–1999	Case series
Bagan et al. [11]	2003	30	-	Case control
Bagan et al. [12]	2004	19	-	Case series
Gandolfo et al. [13]	2009	47	1981–2006	Retrospective case control
Poveda-Roda et al. [14]	2010	17	-	Preliminary study
Gouvea et al. [15]	2013	21	-	Retrospective case control
Garcìa-Chìas et al. [16]	2014	40	1984–2011	Retrospective case series
Flores et al. [17]	2016	15	-	Case control
Garcìa-Pola et al. [18]	2016	14	1984–2015	Observational descriptive study
Borgna et al. [19]	2016	48	1990–2015	Retrospective case series
Thomson et al. [20]	2018	80	1996–2014	Retrospective cohort
Upadhyaya et al. [21]	2018	20	1994–2016	Retrospective case series
Villa et al. [5]	2018	42	1996–2016	Retrospective case series
Bagan et al. [22]	2019	63	2003–2018	Retrospective observational clinical study
Koh et al. [23]	2019	10	-	Case control
Favia et al. [24]	2020	75	1989–2008	Retrospective case series
Llorens et al. [25]	2020	10	-	Retrospective case control
McParland and Warnakulasuriya [26]	2020	51	-	Retrospective case series

**Table 2:** Evaluation of recommendation and quality of evidence of included studies according to Robinson et al. [27]. (2A: weak recommendation; limited quality, patient-oriented evidence. B: Systematic review/meta-analysis of lower quality cohort studies with inconsistent results that may vary depending on circumstances or patients or societal values; retrospective cohort studies; case-control study. C: Consensus guidelines, usual practice, expert opinion, case series; other alternatives may be equally reasonable).

Authors	Year of Publication	Grade of Recommendation	Quality of Evidence
Hansen et al. [2]	1985	2A	C
Zakrzewska et al. [7]	1996	2A	C
Silverman and Gorsky [8]	1997	2A	C
Schoelch et al. [9]	1999	2A	C
Fettig et al. [10]	2000	2A	C
Bagan et al. [11]	2003	2A	B
Bagan et al. [12]	2004	2A	C
Gandolfo et al. [13]	2009	2A	B
Poveda-Roda et al. [14]	2010	2A	B
Gouvea et al. [15]	2013	2A	B
Garcìa-Chìas et al. [16]	2014	2A	C
Flores et al. [17]	2016	2A	B
Garcìa-Pola et al. [18]	2016	2A	C
Borgna et al. [19]	2016	2A	C
Thomson et al. [20]	2018	2A	B
Upadhyaya et al. [21]	2018	2A	C
Villa et al. [5]	2018	2A	C
Bagan et al. [22]	2019	2A	C
Koh et al. [23]	2019	2A	B
Favia et al. [24]	2020	2A	C
Llorens et al. [25]	2020	2A	B
McParland and Warnakulasuriya [26]	2020	2A	C

**Table 3:** Data relating to the number of PVL patients, sex, tobacco consumption, mean follow-up, number of patients with neoplastic evolution of PVL and with development of multiple oral carcinomas.

Authors	Number of PVL Patients	M	F	Tobacco Use	Mean Follow-Up (Y)	Number of PVL Patients with Malignant Transformation	Number of Patients with Multiple Oral Cancers
Hansen 1985 [2]	30	6	24	18	6.1	26/30	-
Zakrzewska 1996 [7]	10	5	5	7	6.6	10/10	-
Silverman 1997 [8]	54	11	43	17	11.6	38/54	12/38
Schoelch 1999 [9]	12	4	8	5	4	2/12	-
Fettig 2000 [10]	10	6	4	3	4.4	6/10	-
Bagan 2003/2004 [11,12]	30	6	24	7	4.7	27/30	10/19
Gandolfo 2009 [13]	47	10	37	17	6.8	19/47	8/19
Poveda-Roda 2010 [14]	17	7	10	6	6.6	5/17	3/5
Gouvea 2013 [15]	21	3	18	4	7.3	9/21	2/9
Garcìa-Chìas 2014 [16]	40	15	25	13	3.6	7/40	-
Flores 2016 [17]	15	0	15	0	5.4	4/15	2/4
Garcìa-Pola 2016 [18]	14	3	11	3	14.5	4/14	-
Borgna 2016 [19]	48	24	24	33	4.3	23/48	-
Thomson 2018 [20]	80	41	39	-	4	2/80	-
Upadhyaya 2018 [21]	20	6	14	12	7.6	9/20	3/9
Villa 2018 [5]	42	7	35	17	4.5	30/42	10/30
Bagan 2019 [22]	63	20	43	25	6 (median)	30/63	-
Koh 2019 [23]	10	5	5	6	3.3	4/10	-
Favia 2020 [24]	75	-	-	-	5.2 (median)	48/75	33/48
Llorens 2020 [25]	10	4	6	3	12.2	6/10	4/6
McParland 2020 [26]	51	25	26	22	16	11/51	-
Total	699	208	416	218/544	7.2	320/699	87/187
